# Thyroid Transcription Factor-1: Structure, Expression, Function and Its Relationship with Disease

**DOI:** 10.1155/2021/9957209

**Published:** 2021-09-28

**Authors:** Lian Guan, Xu Zhao, Lin Tang, Jing Chen, Juanjuan Zhao, Mengmeng Guo, Chao Chen, Ya Zhou, Lin Xu

**Affiliations:** ^1^Key Laboratory of Gene Detection and Therapy of Guizhou Province, Guizhou Zunyi, 563000, China; ^2^Department of Immunology, Zunyi Medical University, Guizhou Zunyi, 563000, China; ^3^Department of Medical Physics, Zunyi Medical University, Guizhou Zunyi, 563000, China

## Abstract

Thyroid transcription factor-1 (TTF-1/NKx2.1) is a member of the NKx2 tissue-specific transcription factor family, which is expressed in thyroid follicle, parathyroid gland, alveolar epithelium, and diencephalon which originated from ectoderm, and participates in the differentiation, development, and functional maintenance of the above organs. Recent studies have shown that the abnormal expression of TTF-1 is closely related to the occurrence of a variety of human diseases and can be used as a potential new target for the diagnosis and treatment of related diseases. In this article, in order to strengthen the systematic understanding of TTF-1 and promote the progress of related research, we reviewed the structure, expression regulation, biological functions of TTF-1, and its role in the occurrence and development of human-related clinical diseases. Meanwhile, we prospect the future research direction of TTF-1, which might ultimately contribute to the understanding of the pathogenesis of related clinical diseases and the development of new prevention and treatment strategies.

## 1. Introduction

**Figure 1:**
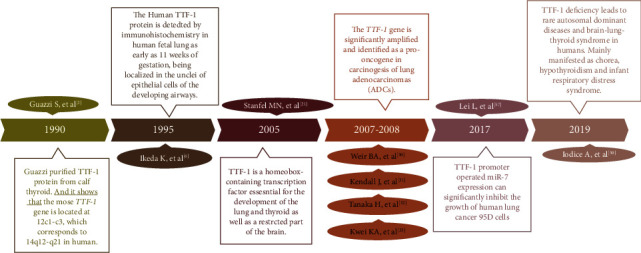
The research and development process of TTF-1.

Thyroid transcription factor 1 (TTF-1) is a tissue-specific transcription factor with homeodomain protein folding structure, which is mainly expressed in differentiated cells derived from the foregut endoderm and neuroectoderm, including thyroid follicular cells and type II alveolar epithelial cells, and regulates the expression of relevant functional genes in the thyroid and lung tissues, promotes the development and differentiation of embryonic, and plays a key role in maintaining the normal function of terminal respiratory unit cells, especially as one of the molecular markers for the diagnosis of thyroid and lung tumors, indicating its good application value in the clinical differential diagnosis of thyroid or lung tumors [1, 2]. Recent studies have also revealed that TTF-1 is also expressed in the pituitary gland, hypothalamus, and other diencephalic tissues, which participates in the development and biological function of the brain tissue, reflecting the complexity of its biological function (Figure 1).

## 2. The Gene Location and Structure of TTF-1

**Figure 2:**
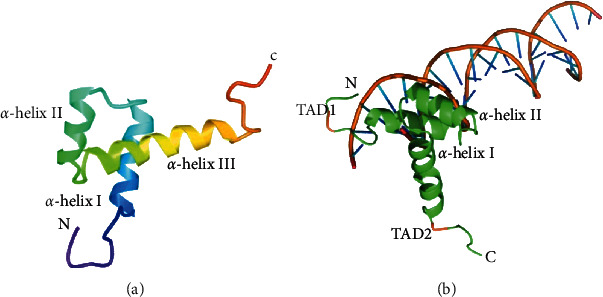
Structure diagram of TTF-1. (a) The homology domain structure diagram of TTF-1. (b) Transcriptional active domains (TAD) and the model structure of the DNA-binding domain (DBD) of the TTF-1.

Thyroid transcription factor-1 (TTF-1), also known as thyroid-specific enhancer binding protein (NKx2.1), is one of the homologous transcription factors in the NKx2 gene family. In 1990, Guazzi [3] purified TTF-1 protein from the calf thyroid, for the first time, and obtained partial amino acid sequence, and the cDNA of TTF-1 was cloned from the calf thyroid cDNA library. In mice, the *TTF-1* gene is located in the C1-C3 region of chromosome 12, which is equivalent to the q12-q21 region of human chromosome 14. The human *TTF-1* gene contains a homeobox region and a 17-amino acid encoding motif which is unique to the NKx family of transcription factors and encodes a nuclear protein with a relative molecular mass of 38,000. Since the TTF-1 gene was first discovered as a thyroid-specific DNA functional structure that can interact with rat thyroglobulin, it was named thyroid transcription factor. The TTF-1 protein contains a highly conserved sequence of 371 amino acid polypeptides with a three-dimensional structure containing 1 DNA binding domain (DBD) and 2 transcriptional active domains (TAD). The DNA binding domain is a HD structure composed of three helices which consist of 60 amino acid residues. Helix I and II are combined with the carbon skeleton of DNA, and helix III is highly conserved in evolution and can recognize specific DNA sequences, in which the core of the sequence is 5′ -CAAG-3. [4–8] Among the two TADs of TTF-1 protein, one is located between the 51 and 123th amino acid residues at the N-terminal, and the other is located between the 295 and 372th amino acid residues at the C-terminal, both of which can bind to DNA **(**Figure 2**)**. In a hydrophilic environment, the N-terminus is a transcriptionally active region, whereas in a hydrophobic environment, it can induce the loss of binding function by forming an alpha helix formation [9], thereby regulating the transcription of a variety of thyroid-specific genes and lung genes.

## 3. The Expression of TTF-1

The expression of TTF-1 is strictly regulated during the period of embryonic development. The expression of TTF-1 appeared earliest in the early foregut endoderm and then was abundantly expressed in tracheal precursor cells generated from the lung primordia. In the lung, TTF-1 protein was detected in fetal lungs as early as 11 weeks of gestation, which is located in the nucleus of the columnar epithelial cell of the lung bud derived from the endoderm, and regulates the expression of surfactant protein and Clara cell secretory protein. Before 16 weeks of human embryonic development, TTF-1 is most abundantly expressed in distal airways compared with proximal bronchial epithelial cells, especially around lobules and adjacent distal airways. At 19 weeks of gestation, TTF-1 was strongly expressed in many epithelial cell nuclei of the new bronchial buds. At 20-24 weeks, TTF-1-positive cells were mainly distributed in the bronchioles around the pulmonary lobules, while the expression of TTF-1 in the tracheal epithelial cells gradually weakened after 25 weeks of embryonic development, especially in the third trimester. At 36 weeks of gestation, the expression of TTF-1 in all levels of airways decreased, while the nuclei of respiratory tract and alveolar type II cells still maintained expression [10]. After birth, TTF-1 was stably expressed in type II alveolar cells. In the thyroid gland, TTF-1 is expressed during thyroid formation and migration during embryonic development and earlier than the expression of genes related to thyroid follicular cell differentiation such as thyroglobulin (TG), thyroid peroxidase (TPO), and thyrotropin receptor (TSHR), suggesting that TTF-1 is critical for early thyroid development. In addition, TTF-1 can also be expressed in the ventral forebrain, diencephalon, nearby telencephalon, and hypothalamus during embryonic development. As a transcription factor of homologous structure necessary for their normal development, TTF-1 is still slightly expressed in the hypothalamus after birth and plays an important role in sexual development [11].

## 4. The Regulation of TTF-1 Expression

### 4.1. Regulation of Transcription Factors

**Figure 3:**
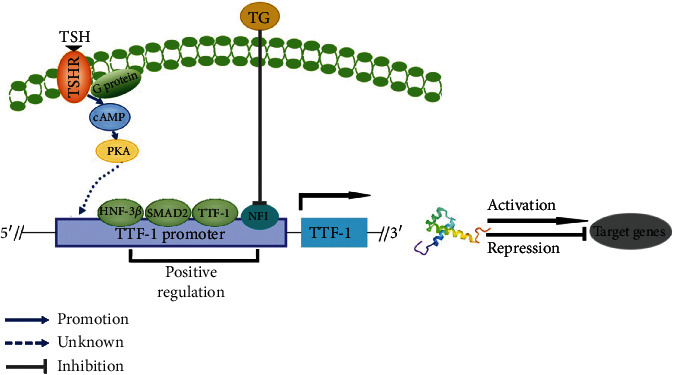
Regulatory mechanisms of NKx2-1/TTF-1 expression. The expression of TTF-1 is regulated by thyroid-stimulating hormone (TSH), nuclear factor (NFI), and the binding site of TTF-1 itself; follicular thyroglobulin (TG) can reduce the level of NFI mRNA and its protein (especially the level of NFI-A protein) and reduce the binding of TTF-1 and NFI to regulate the expression of TTF-1. In addition, when TSH binds to the thyroid-stimulating hormone receptor on the cell surface, it couples with the alpha subunit of the G protein on the cell surface and activates cAMP through the G protein mediation to activate PKA to increase TTF-1 expression. Moreover, TTF-1 can activate its expression to form a positive feedback. The transcription factors that interact with TTF-1 such as Smad2 and HNF3*β* can regulate the transcription activity of TTF-1.

The regulatory region of TTF-1 gene expression has CRE elements (located on the TSHR gene promoter) regulated by thyroid-stimulating hormone (TSH), nuclear factor (NFI), and the binding site of TTF-1 itself. Nakazato et al. [12] found that there are two NFI sites in -233 to -202 BP and -192 to -153 BP of the main transcription start site in the proximal 5′- terminal region of the TTF-1 gene. These two NFI sites enhance the expression and function of TTF-1 gene by binding to four major NFI proteins (NFI-A, NFI-B, NFI-C, and NFI-X) in functional thyroid cells, while the absence of NFI sites decrease TTF-1 expression. Studies have reported that follicular thyroglobulin (TG) can reduce the level of NFI mRNA and its protein levels (especially the NFI-A protein) and reduce the binding of TTF-1 and NFI to regulate the expression of TTF-1, and TG can also inhibit the expression of TTF-1 through the oxidation-reduction method by reducing its binding activity. In addition, when TSH binds to the thyroid-stimulating hormone receptor on the cell surface, it couples with the alpha subunit of the G protein on the cell surface, leading the activation of cAMP through the G protein mediation to activate PKA. After PKA activation, it further activates the nuclear TTF-1 to increase its expression. In addition, TTF-1 can activate its expression itself through two binding sites of TTF-1 in the thyrotropin receptor, in the condition of hyperthyroid, to form a positive feedback to achieve autoregulation of TTF-1 [13] (Figure 3).

Furthermore, some recent evidence has shown that transcription factors interacting with TTF-1 also include HNF-3*β*, Smad2, FOXA1, FOXP2, and GATA6, which regulate the transcriptional activity of TTF-1 in lung epithelial cells, respectively [14–19]. Finally, CHIP-seq analysis also identified other potential transcriptional targets of TTF-1, among which LMO3, E2F3, and cyclins B1 and B2 induce TTF-1 expression, while MUC5A, FGFR1, and MET inhibit its expression [20]. Thus, these accumulated studies point to the complexity of the regulation mechanism of TTF-1 expression.

### 4.2. Regulation of Covalent Modification

The covalent modification of phosphorylation-dephosphorylation and glycosylation are also critical for the expression regulation mechanism of TTF-1. As mentioned above, the TSH\cAMP signal pathway activates adenylate cyclase to activate protein kinase A (PKA), which targets the seven serine residues of TTF-1, and the phosphorylation of serine enhances the transcriptional activity of TTF-1. In addition, studies have also found that DTG (a DNA repair enzyme that mediates *trans*-glycosylation regulation) can inhibit the transcription activity of TTF-1 by acting on the nonhomologous domain at the carboxyl terminal of TTF-1. Ultimately, oxidation-reduction regulation can also interfere with the proper folding of the homeodomain of TTF-1 and thereby modulate its DNA binding activity, affecting the expression of TTF-1 itself [21].

## 5. The Biological Function of TTF-1

TTF-1 is mainly involved in the regulation of thyroid-specific genes, as well as the activation of lung surfactant and pituitary genes, which are mainly mediated by transcription factors such as fibroblast growth factor, bone morphogenetic protein, and sonic hedgehog factor. TTF -1 plays a vital role in the development and maturation of the thyroid, lung bronchus, and central nervous system [22]. During embryonic lung development, thyroid transcription factor-1 (TTF-1) plays an important role in lung morphogenesis, differentiation of pulmonary epithelial cells, transcription of surfactant proteins (SP-A, SP-B, and SP-C), and secreted proteins from C Lara cells. Studies have shown that TTF-1 expression is significantly increased in the early (E11.5) and late (E19.5) stages of lung development, which directly regulates cell cycle effectors related to lung development, while the absence of TTF-1 can affect cell cycle progression, which in turn affects the lung development process [23].

In the process of thyroid development, TTF-1 and other thyroid transcription factors (PAX8, FOXE1, and HHEX) are coexpressed in thyroid precursor cells and thyroid follicular cells, and its encoding genes are continuously expressed in mature thyroid cells, thereby establishing and maintaining the thyroid phenotype, function, homeostasis, and tissue differentiation of the thyroid gland. However, in the absence of TTF-1, thyroid precursor cells undergo apoptosis and disappear in the early stage of embryonic development (E10.5 ~ 11.5), leading to the degeneration of the thyroid, the reduction of the thyroid follicular glial, and abnormal development of mature thyroid tissue [24].

Furthermore, TTF-1 is expressed in the pituitary, hypothalamus, and other diencephalic tissues and participates in the normal development of brain tissue. Kimura et al. [25] observed the defect in the ventral forebrain and pituitary in TTF-1 knockout mice. Sandberg et al. [26] further found that TTF-1 could regulate the permissive chromatin state and transcriptional activation in the subventricular and mantle zones and control the development of interneurons in the ventricular zone. Furthermore, TTF-1 is indispensable not only for the development of parts of the ventral forebrain, such as the median ganglion eminence (MGE) and the preoptic area (POA), but also for the generation of TTF-1-derived cell lineages, including GABAergic neurons, NG2 glia (or oligodendrocytes), and astrocytes [27]. Among them, the absence of NG2 glial cells severely affects vascular development in all telencephalons, resulting in a decrease in branching and connecting blood vessels [28]. Meanwhile, in TTF-1 knockout mice, the decrease in the number of GABAergic neurons and the loss of astrocytes also resulted in minor axonal branching and growth defects in the corpus callosum. In addition, the mechanism by which TTF-1 regulates the production of astrocytes is related to its regulation of the proliferation of precursor cells. Studies have shown that TTF-1 can bind to the promoter of glial fibrillary acidic protein (GFAP), which mainly expresses in astrocytes, to regulate its expression and affect the production of astrocytes [29]. Ultimately, recent studies have also found that there are a large number of TTF-1 binding sites in the 5′ side of hemeoxygenase-1 gene (HO-1, an anti-inflammatory cytoprotective enzyme), and TTF-1 can affect HO-1 transcription in the mouse hypothalamus and astrocytes and thereby participating in the regulation of TNF-*α*-mediated inflammatory response in the mouse hypothalamus [30].

## 6. The Relationship between TTF-1 and Human Diseases

### 6.1. TTF-1 and Lung cancer

A large number of studies have shown that TTF-1 plays a vital role in the occurrence of lung cancer. It was found that there is a significant amplification on TTF-1 gene locus in lung adenocarcinoma (ADCs), leading to increased proliferation and viability of lung cancer cells [31–34]. TTF-1 is expressed in nearly 75% of nonmucinous lung adenocarcinoma. However, lung squamous cell carcinoma does not express TTF-1, which is most commonly used to distinguish primary lung adenocarcinoma from other metastatic tumors, suggesting its important application value in the diagnosis and differential diagnosis of lung cancer [35].. Moreover, the positive ratio of TTF-1 expression is closely related to the differentiation degree of ADC tumor tissue. In terms of mechanism, studies have shown that TTF-1 can regulate the growth and metastasis of lung cancer cells through a variety of downstream target genes, including Selenbp1, EGFR, Foxa2, CDX2, and DDB1. [36–40]. Yamaguchi et al. [41] further identified ROR1 as a direct transcriptional target of TTF-1, which induces receptor tyrosine kinase like receptor expression and ERBB3 phosphorylation via ROR1 kinase-dependent c-Src activation, maintaining the balance between the prosurvival PI3K-Akt and proapoptotic p38 signaling pathways. In virtue of the possibility of TTF-1 as a diagnostic or prognostic marker, a large number of retrospective studies have been conducted on the prognosis of non-small-cell lung cancer (NSCLC), especially lung adenocarcinoma. Puglisi et al. [42] found that the expression of TTF-1 was negatively correlated with the prognosis of patients with lung adenocarcinoma. Anagnostou et al. [43] found that the survival ratio of patients with stage I lung adenocarcinoma was positively correlated with the expression degree of TTF-1. Furthermore, Takeuchi et al. [44] retrospectively analyzed 82 patients with nonsquamous cellcarcinoma NSCLC who received standard monotherapy of docetaxel (a standard second/third-line treatments for non-small-cell lung cancer after a failed chemotherapy response). It was found that the disease control ratio of TTF-1-positive patients and TTF-1-negative patients were 69% and 42%, respectively, and the median survival time was 393 days and 221.5 days, respectively. In addition, the progression-free survival time of TTF-1-positive patients tended to be longer. However, on multivariate analysis, it showed that TTF-1 positive is a significant predictor of overall survival after docetaxel monotherapy. Therefore, TTF-1 is currently of great value in the diagnosis and differential diagnosis of lung cancer, but its possibility as a prognostic marker needs further research to be clarified. Furthermore, TTF-1 is also associated with the occurrence of small-cell lung cancer (SCLC). It was found that the TTF-1 was upregulated in SCLC. Horie et al. [45] further studies showed that ASCL1, as an important transcription factor of neuroendocrine differentiation, regulates the growth and metastasis of SCLC cells through the TTF-1/NFIB axis. Hokari et al. [46] found that TTF-1 participates in regulating the expression of the Bcl-2 gene family in SCLC and showed an antiapoptotic effect. There are few studies on the prognostic value of TTF-1 in small-cell lung cancer (SCLC). However, Wang et al. [47] explored the relationship between the TTF-1 expression status and sensitivity to first-line chemotherapy and prognosis of SCLC patients; analysis of 243 patients revealed that the objective response rate (ORR), median progression-free survival (PFS), and median overall survival (OS) of first-line chemotherapy in patients with positive expression of TTF-1 was higher than that in patients with negative expression of TTF-1, suggesting that TTF-1 may serve as a biomarker for predicting efficacy and prognosis in SCLC. However, the mechanism of TTF-1 in regulating the occurrence of different types of lung cancer remains to be further explored.

Interestingly, some studies have shown that TTF-1 can inhibit the growth of lung cancer and plays a double-edged role in the occurrence and development of lung cancer [48, 49]. Winslow et al. [50] injected the lentiviral vectors expressing Cre-recombinase into a mouse model which harbours an activating point in KRAS and inactivation of P53. Cross-species analysis and the function experiments identified that the TTF-1 inhibits the differentiation and metastatic potential of lung cancer *in vivo*. Furthermore, Maeda et al. [51] induced oncogenic KrasG12D in the respiratory epithelium of TTF-1 heterozygous (TTF-1 +/-) mice and found that decreased expression of TTF-1 promotes the initiation and progression of aggressive KrasG12D-induced mucinous lung adenocarcinoma. Besides, the deletion of TTF-1 seems to induce the phenotype of mucin through the subsequent release of Foxa1/Foxa2, and human lung infiltrating mucinous adenocarcinomas almost always express HNF4A and have a significant connection with negative TTF-1 expression and positive KRAS mutation status [52]. Therefore, these studies suggested that the complex biological function of TTF-1 in the development of lung cancer and the exact mechanism remains to be fully elucidated.

### 6.2. TTF-1 and Thyroid Disease

TTF-1 is the main factor that promotes the differentiation, development, and proliferation of the thyroid during the embryonic stage and is also responsible for maintaining the normal function of the thyroid after birth. Congenital hypothyroidism (CH), a common endocrine and metabolic disease in the neonatal period, is a physical and intellectual developmental disorder caused by a low metabolic level resulting from insufficient synthesis and secretion of thyroid hormones. Accumulating evidences have shown that about 85% of patients are caused by abnormal development of the thyroid. More importantly, the mutation of TTF-1 gene is an important factor in causing the disease. The mutations of TTF-1 gene mostly involve the homologous domain of the gene, including insertion mutation, heterozygous mutation, and deletion mutation. The mutant TTF-1 cannot bind to the target DNA, thereby losing its regulatory function, affecting the normal differentiation and maturation of thyroid cells, and even causing malignant transformation, leading to the occurrence of thyroid cancer. Among them, missense mutations in the TTF-1 gene resulting in alanine to valine substitution at codon 339 have been identified in families with multinodular goiter and papillary thyroid cancer [53]. Gudmundsson et al. [54] also found that the SNPrs944289 gene was significantly associated with increased risk of thyroid cancer, but the specific mechanism between SNPrs944289 and TTF-1 gene remains unclear. Dupain et al. [55] also found that both TTF-1 and Pax-8 have antiproliferation and antitumorigenic properties when they are coexpressed. However, when their expression is above the threshold level (especially the abnormal expression of TTF-1), they can induce the occurrence of thyroid tumors. As the TTF-1 expression decreases with tumor differentiation from benign thyroid tumor, follicular thyroid carcinoma, and undifferentiated carcinoma, TTF-1 can be used as a prognostic indicator for judging the malignancy of thyroid tumors. In addition, studies have shown that TTF-1 may lead to the occurrence of thyroid autoimmune diseases by regulating the expression of MHC-I molecules. MHC-I molecules are involved in the initiation and pathological process of thyroid autoimmune diseases and thyroid tumors, while TTF-1 is the main factor regulating MHC-I molecules. Consequently, the abnormal regulation of TTF-1 on MHC-I molecules may be an important cause for the pathogenesis [56]. Therefore, it necessitates the study of the regulatory mechanism of TTF-1 on MHC-I and the interaction between TTF-1 and MHC-I in different disease states for the treatment on thyroid-related diseases.

### 6.3. TTF-1 and Genetic Diseases

TTF-1 deficiency leads to rare autosomal dominant diseases and brain-lung-thyroid syndrome in humans, such as chorea, hypothyroidism, and infant respiratory distress syndrome. Benign hereditary chorea (BHC) is an inherited disease of autosomal dominant dyskinesia, which is caused by brain development disorders. Symptoms appear in the early stage (usually before the age of 5), and some BHC family patients tend to slow down in adulthood. BHC is nonprogressive compared to Huntington's disease; patients have normal or slightly subnormal intelligence and mildly show slight jitter of muscles and choreiform movements. It has been confirmed that there is a BHC gene locus on human chromosome 14, a detailed analysis of its key regions, a complete deletion of the 1.2 Mb region containing the TTF-1 gene was found [57]. Meanwhile, chorea, with mutations in the TTF-1 gene, was defined as a predominant upper limb movement disorder and improved with age. All patients presented with clinical or subclinical hypothyroidism, specifically decreased serum ferritin levels that can lead to restless legs syndrome- (RIS-) like symptoms [58]. More importantly, all patients were heterozygous mutations, suggesting a dominant inheritance pattern of the disease.

**Figure 4:**
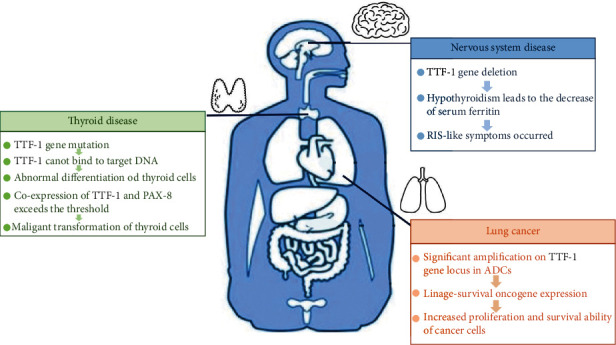
TTF-1 is involved in the pathogenesis of lung cancer, thyroid diseases, and neurological diseases. In the lungs, the significant amplification on TTF-1 gene locus in lung adenocarcinoma (ADCs) leading to the enhancement of lung cancer cell proliferation and survival ability. In the thyroid, the mutations of the TTF-1 gene leading the TTF-1 cannot bind to the target DNA, thereby losing its regulatory function, affecting the normal differentiation and maturation of thyroid cells, and even causing malignant transformation. In the brain, patients with TTF-1 gene mutation, chorea mainly manifested as upper limb dyskinesia. All patients showed clinical or subclinical hypothyroidism, especially the decrease of serum ferritin level, which could lead to restless legs syndrome (RIS) like symptom.

Besides, BHC patients with TTF-1 mutations present with primary hypothyroidism, respiratory distress, and neurological symptoms, showing a cerebropulmonary thyroid syndrome [59, 60].. Das et al. [61] found that in a large proportion of neonates who died due to respiratory disease, of whom neonatal hyaline membrane disease (HMD) was the most common, TTF-1 expression was significantly reduced in the distal airways and alveoli in HMD. In addition, mutations in the TTF-1 gene can also affect the normal development of the striatum, resulting in the reduction of acetylcholine synthase, acetylcholine transferase, dopamine, and acetylcholine receptors in the striatum, which may be related to the pathogenesis of BHC. Invernizzi et al. [62] also found that the deletion of the MBIP gene (which is believed to be involved in the pathogenesis of lung-brain-thyroid syndrome) can cause mutations in the TTF-1 gene, which significantly affects the expression of TTF-1 **(**Figure 4**)**.

## 7. Conclusions and Future Perspectives

**Figure 5:**
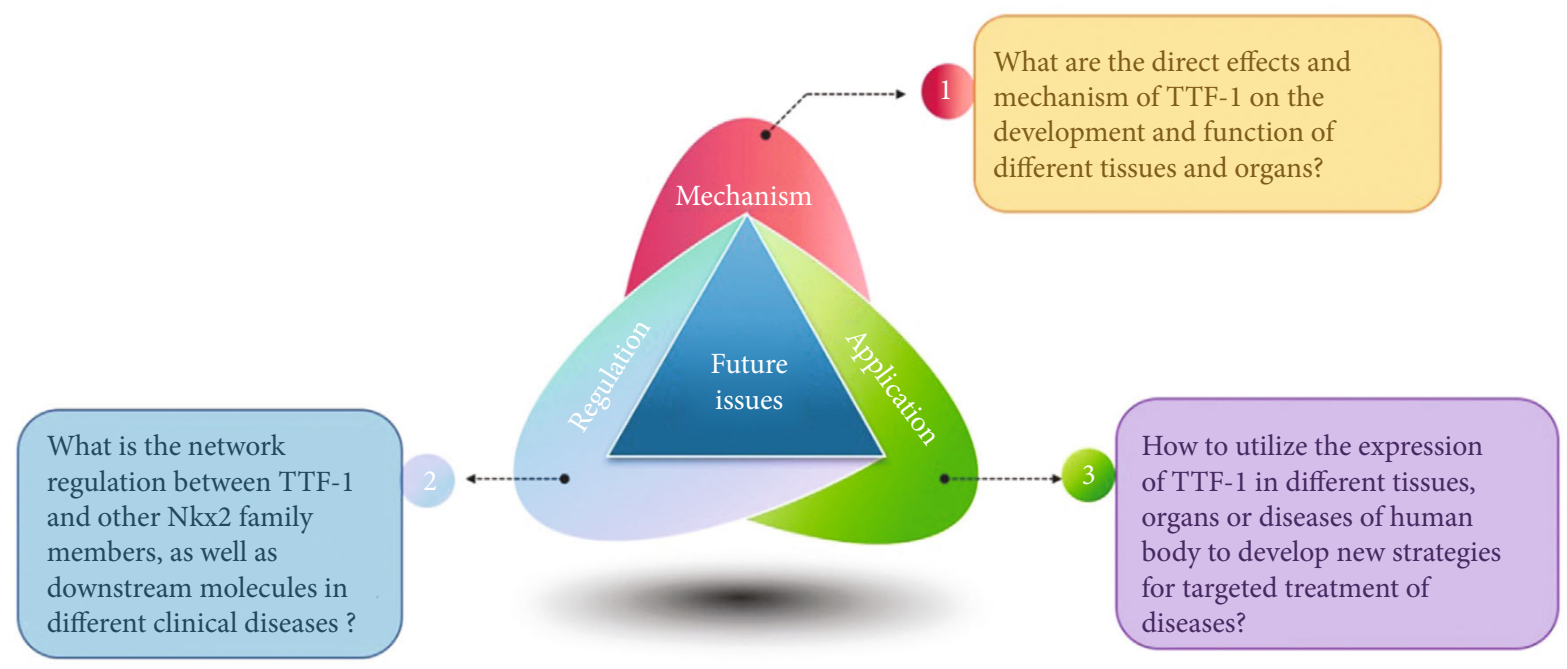
A sketch of scientific issues of the future exploration of TTF-1. Currently, there are still many unclear scientific issues, belonging to basic and applied research fields, on the role of TTF-1 in the development of organs and related diseases.

Existing studies have shown that TTF-1 is a member of the thyroid tissue-specific transcription factor family. Its expression and deletion are closely related to the development of the thyroid, lung, and other tissues, as well as the occurrence of a variety of clinical diseases such as tumors, thyroid diseases, and nervous system diseases [63, 64]. A large amount of emerging evidence has drawn attention to its role in cancer. The latest research shows that TFF-1 is strongly expressed in non-TRU lung adenocarcinomas with gastrointestinal characteristics and is associated with a poorer prognosis in advanced stages. Knockdown of TFF-1 inhibits the proliferation of cancer cells, indicating that TFF-1 is a potential target for cancer treatment of nonauthentic lung adenocarcinoma [65]. And it has an important potential value in the diagnosis and treatment of related diseases [66, 67]. Recent research shows PD-L1/TTF-1 double immunohistochemistry can be successfully applied to cytopathological specimens to better identify patients who may benefit from immune checkpoint blockade therapy [68]. Furthermore, studies have also shown that the TTF-1 promoter-operated miR-7 expression can significantly inhibit the growth of human lung cancer 95D cells [69], indicating the potential value of TTF-1 in cancer gene therapy. However, due to the complexity of TTF-1 transcriptional regulation and downstream molecules, there are still a lot of scientific problems need to be solved urgently, such as what are the direct effects and mechanism of TTF-1 on the development and function of different tissues and organs, what is the network regulation between TTF-1 and other NKx2 family members, as well as downstream molecules in different clinical diseases, and how to utilize the expression of TTF-1 in different tissues, organs, or diseases of the human body to develop new strategies for targeted treatment of diseases (Figure 5). With the continuous development of biotechnology and bioinformatics technology, we believe that more in-depth study of the TTF-1 gene will not only contribute to a more complete understanding of the network regulatory mechanisms of NKx family molecules represented by TTF-1 but also have important significance for the development of new strategies for clinical disease prevention and treatment.

## Data Availability

Not applicable.

## Abbreviations

TTF-1: Thyroid transcription factor-1
NKx2-1: NK2 homeobox 1
DBD: DNA binding domain
TAD: Transcriptional active domain
TG: Thyroglobulin
TPO: Thyroid peroxidase
TSHR: Thyrotropin receptor
TSH: Thyroid-stimulating hormone
NFI: Nuclear factor
cAMP: Cyclic adenosine monophosphate
PKA: Protein kinase a system
FOXA2: Forkhead box A2
FOXP2: Forkhead box p2
GATA6: GATA binding protein 6
STAT3: Signal transducer and activator of transcription 3
RAR: Retinol receptor
LOM3: LIM domain only 3
FGFR1: Fibroblast growth factor receptor-1
DTG: DNA *trans*-glycosylation
MGE: Median ganglion eminence
POA: Preoptic area
GABA: *γ*-Aminobutyric acid
GFAP: Glial fibrillary acidic protein
HO-1: Hemeoxygenase-1
TNF: Tumor necrosis factor
ADCs: Adenocarcinoma
NSCLC: Non-small-cell lung cancer
SCLC: Small-cell lung cancer
ORR: Objective response rate
PFS: Median progression-free survival
OS: Overall survival
CH: Congenital hypothyroidism
BHC: Benign hereditary chorea
RIS: Restless legs syndrome
HMD: Hyaline membrane disease.
